# Scanning Gas Diffusion Electrode Setup for Real-Time Analysis of Catalyst Layers

**DOI:** 10.1021/acsmeasuresciau.4c00018

**Published:** 2024-07-12

**Authors:** Ina Reichmann, Vicent Lloret, Konrad Ehelebe, Pascal Lauf, Ken Jenewein, Karl J. J. Mayrhofer, Serhiy Cherevko

**Affiliations:** † Forschungzentrum Jülich GmbH, Helmholtz Institute for Renewable Energy (IET-2), Cauerstraße 1, Erlangen 91058, Germany; ‡ Friedrich-Alexander-University Erlangen, Nürnberg, Erlangen 91058, Germany

**Keywords:** fuel cell, electrocatalysis, gas-diffusion-electrode, oxygen reduction reaction, stability, platinum, ICP-MS

## Abstract

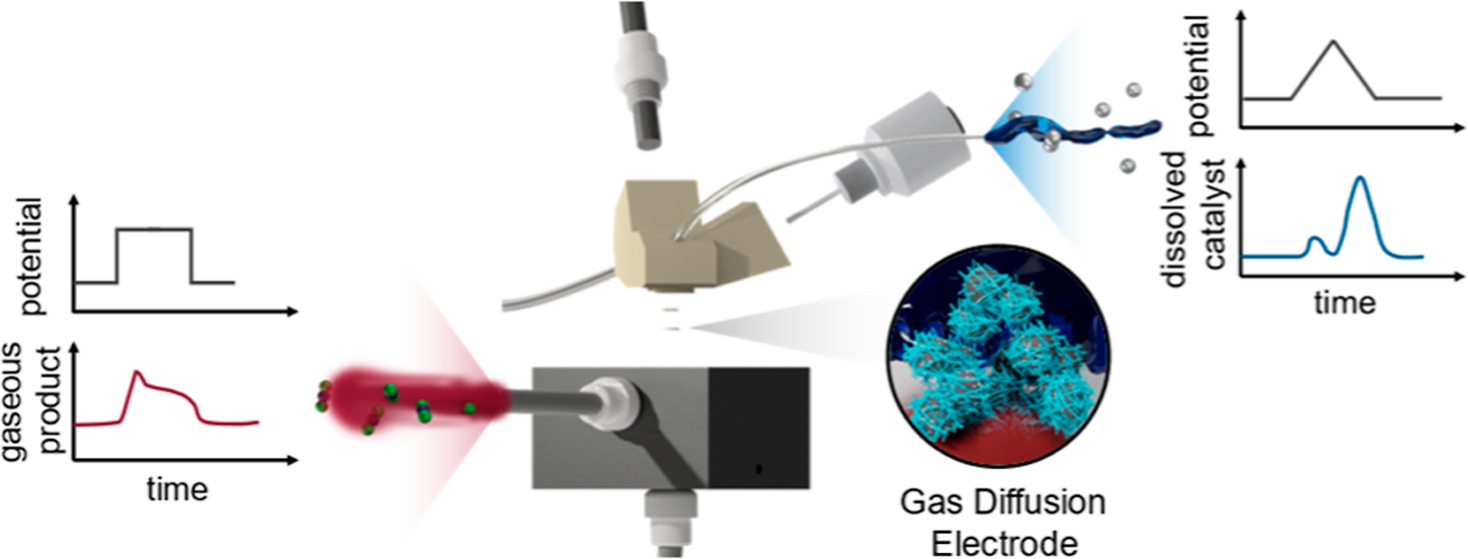

The scanning gas diffusion electrode (S-GDE) half-cell is introduced as a new tool to improve the evaluation of electrodes used in electrochemical energy conversion technologies. It allows both fast screening and fundamental studies of real catalyst layers by applying coupled mass spectrometry techniques such as inductively coupled plasma mass spectrometry and online gas mass spectrometry. Hence, the proposed setup overcomes the limitations of aqueous model systems and full cell-level studies, bridging the gap between the two approaches. In this proof-of-concept work, standard fuel cell electrodes are investigated at elevated oxygen reduction reaction current densities, while dissolved Pt^*x*+^ ions in the electrolyte and gaseous CO_2_ in the outlet gas stream are detected to track platinum dissolution and carbon corrosion, respectively. Relevant current densities of up to 0.75 A cm^–2^ are demonstrated. The electrochemically active surface area, oxygen reduction reaction activity, and Pt dissolution rates are quantified and benchmarked to the values obtained in the conventional stationary GDE half-cell. Moreover, it is found that Pt dissolution is suppressed when O_2_ is purged into the catalyst layer. Overall, this work demonstrates the feasibility of fast fuel cell electrode screening obtaining, complementary to electrochemical, mass spectrometry data necessary in fundamental studies on structure/performance relationships under actual reaction conditions. While Pt/C, in relevance to its fuel cell application, is used in this study, the proposed setup can be applied in water electrolysis, CO_2_ conversion, metal-air batteries, and other neighbor technologies.

## 1 Introduction

Global emissions have to be drastically reduced in order to meet the two-degree Celsius target of the Paris Agreement^1^ necessitating a profound transformation of our current energy structure. Fossil fuels-based technologies must be replaced by energy conversion and storage processes relying on emission-free green energy sources, such as fuel cells and batteries. Furthermore, the production of commodity chemicals derived from crude oil will also drastically change, given the energy consumption of this industry. Fuel cells and electrolyzers used for electricity generation^2^ and commodity chemical synthesis via CO_2_ conversion^3^ seem to be a promising part of the shift in our energy landscape. One of the bottlenecks to the wide-scaled application of fuel cells is the catalyst used in those. Proton exchange membrane fuel cells (PEMFCs), as an example, are suitable for replacing fossil fuel engines in transport vehicles due to their rapid start-up time and good power-to-weight ratios in comparison to other fuel cells.^4^ Yet, platinum (Pt)-based materials seem to be the only viable option as a catalyst for PEMFCs despite decades of enormous research to replace them with a cheaper alternative. Using the noble metal as a catalyst causes tremendous material costs in commercial applications. Despite progress, more research is needed to replace Pt as part of the electrode or lower the loading for widespread utilization of PEMFCs.^5^

Most catalyst testing is conducted in so-called aqueous model systems (AMS), which allow simple and fast electrochemical protocols to assess a catalyst’s stability and activity.^6^ The rotating disk electrode (RDE) half-cell setup has been established as the primary AMS to evaluate new materials due to the small amount of catalyst needed and the defined diffusion layer, facilitating the determination of kinetic data via short electrochemical protocols.^7,8^ As the RDE half-cell setup is a closed system and does not allow fast screening of multiple catalysts, different approaches to scan catalysts have been developed, such as the scanning electrochemical microscopy (SCEM)^9^ or the scanning flow cell (SFC).^10^ SCEM utilizes a small nanoelectrode, typically encapsulated in an insulating body, which can detect free-diffusing species electrochemically. The SCEM can screen whole arrays of different catalyst compositions by applying different electrochemical protocols and quantifying the products, a method that finds application, for example, in the ongoing study for efficient catalysts for the reduction of CO_2_^11^ or battery research.^12^ Another example of an electrochemical scanning technique is the SFC,^10^ which is mainly used to study the electrochemical stability of different catalysts. Due to its adaptable design, the SFC finds application in diverse research areas, such as oxygen evolution reaction,^13,14^ photoelectrochemistry,^15^ and battery research.^16^ Yet, the main advantage of the SFC is its seamless integration of external mass spectrometry such as an inductive coupled plasma-mass spectrometer (ICP-MS) to investigate the stability of a catalyst by quantifying its dissolution^17^ or an online electrochemical mass spectrometer (OLEMS), allowing the analysis of gaseous products.^18^

Nevertheless, AMS half-cells have their limitations, as the environment of the catalyst and the operating conditions in these cells drastically differ from the conditions in a membrane electrode assembly (MEA), which has been extensively discussed in the literature, so only the major points will be summarized. For a more comprehensive discussion, the interested reader is referred to the literature.^6,19,20^ First, AMS half-cells are limited by the gas solubility in the electrolyte, limiting the reachable currents to the kinetic region. Furthermore, the electrode structure differs massively from the electrodes employed in a real fuel cell, affecting their performance.^19^ In contrast to the microporous electrodes used in fuel cells, which ensure a good gas distribution across the catalyst layer, thin drop-casted catalyst layers are typically employed in AMS half-cells. These differences in electrode architecture can affect the catalyst: For example, a membrane on the catalyst creates a milder environment than the liquid electrolyte, causing less catalyst degradation.^21^ When studying the dissolution, it is affected by the catalyst’s microstructure, resulting in lower apparent catalyst dissolving due to enhanced redeposition.^22^ However, not only do the gas availability and catalyst layer structure lead to significant differences, but the operation conditions also affect the electrochemical activity of different catalysts.^19,23,24^ Hence, promising activity and, especially, stability results from RDE and SFC measurements of different catalysts are not always reproducible in MEA systems.^7,25^

Considering the lack of replacements for PGM as ORR catalysts, the focus of the research started to shift from finding a Pt replacement to catalyst layer optimization, such as tuning the ionomer,^26^ or the used carbon supports.^27^ Toyota, as an applied example, enhanced the fuel cell stack in the Mirai fuel cell car series from the first to the second generation by refining the electrode’s conductivity and oxygen permeation while not changing the catalyst. Adapting a mesoporous support increased the stability, and a change in ionomer enhanced the oxygen permeability, boosting mass power density from 2.8 to 5.4 kW kg^–1^ compared to the first generation, along with improvements in the flow field and bipolar plates.^28^

While there are excellent and established methods for fuel cells to assess catalysts, including well-defined protocols and development targets,^29^ these test procedures remain time- and cost-intensive.

Although testing catalysts in an MEA setup mirrors real conditions, these procedures can be time- and cost-intensive. As fuel cell electrodes are normally produced with higher loadings and larger geometrical areas than catalyst layers employed in AMS half-cell setups, higher amounts of costly catalysts are needed. The factors hinder the process of effectively screening novel materials.^19^

Furthermore, coupling a fuel cell to online analysis tools can still be challenging and requires intensive development, depending on the instrument.^30,31^ Hence, a fast analysis of the catalyst performance in an optimized catalyst layer proves challenging.

Since the 1990s, gas diffusion electrode (GDE) half-cell setups have been introduced to investigate fuel cell electrodes in a simplified manner.^32,33^ The GDE half-cell is a three-electrode cell that incorporates catalyst layers with or without membranes, as its working electrode is similar to those used in PEMFC applications. Old designs of this cell used to be simple and most likely suffered from nonoptimized design and flooded electrodes, resulting in low current densities.^34,35^ Recently, thanks to new developments in this area, such as optimized flow fields positioned behind the working electrode allowing for constant gas purging into the working electrode, high current densities, up to 2 mA cm^–2^ without mass transport limitations, were achieved.^36,37^ This unique characteristic differentiates these cells from conventional AMS cells, such as the RDE. GDE setups are used in various research fields, from CO_2_ reduction^38,39^ to electrolysis,^40−42^ zinc-air batteries,^43^ and fuel cells.^44−47^ Different groups adjusted the GDE half-cells to fit the reaction’s condition: While for ORR and OER application, mainly bulk cells are used,^36,40−42,44−48^ and flow cells are utilized for CO_2_ conversion as they decrease mass transport limitations, which can occur during the reaction.^22^

Although it is common to analyze the products of CO_2_ with different external analytics such as gas chromatography or high-pressure liquid chromatography,^39^ only a few publications report on these cells connected to mass spectrometry. Ehelebe et al.^22^ pioneered the GDE setup and ICP-MS coupling approach by investigating the effects of platinum loading and the catalyst-electrolyte interphase on Pt dissolution of realistic fuel cell electrodes. Later, this approach was used to examine the stability of FeNC catalyst at relevant current densities.^47^ Niether et al.^49^ developed a three-electrode cell for high-temperature application and optimized the cell for an OLEMS setup. GDEs could be tested in this cell assembly, employing gaseous reactants to study ethylene oxidation and carbon corrosion.^49^ However, the GDE half-cells utilized in the literature suffer from disadvantages similar to the RDE, lacking the flexibility and full automatization potential for high-throughput applications compared to the previously mentioned SFC.

This study introduces a novel tool that seeks to integrate the benefits of both the SFC and GDE configuration. The scanning GDE (S-GDE) is a small flow cell enabling the online analysis of larger quantities of samples in a shorter time frame. At the same time, the cell is optimized for high current densities aimed at closing the gap between AMS and MEA cells. This work will discuss the improvements of the design and its development and the cell’s electrochemical comparability to already published setups and its compatibility with mass spectrometry. The investigation of a Pt/C catalyst was chosen as a model system, as this catalyst is thoroughly investigated.^22−24,26,27,36,45,48,50−52^ First, the S-GDE has been electrochemically established comparing cyclic voltammograms (CVs), the electrochemical surface area (ECSA), polarization curves in O_2_ and Tafel Plot to the already published cells proving a sound system within the margin of error. Second, the cell was linked to two online mass spectrometers to assess its compatibility with these systems. An investigation into the dissolution of Pt in both argon and oxygen environments was conducted using an online ICP-MS approach. Additionally, a carbon corrosion analysis was performed with the assistance of an OLEMS.^53^

## 2 Experimental Section

### 2.1 Sample Preparation

As in our previous works^22,45,54^ a Pt/C (HiSPEC4000, 40 wt % Pt on Vulcan) GDEs have been prepared in-house with an ultrasonic spray coater. The ink consisted of 20 wt % isopropyl alcohol and 30 wt % ionomer. The ink was spray coated on a gas diffusion media (Freudenberg H23C8) to achieve a loading of 0.1 mg cm^–2^ for the electrochemical evaluation and 0.12 mg cm^–2^ for the mass spectrometry measurements (OLEMS and ICP-MS, respectively).

### 2.2 S-GDE Coupled to ICP-MS and OLEMS

The S-GDE is mounted above the flow field with a force sensor (ME Messinstrumente GmbH, KD45 50N/VA/HT), controlling the contact pressure of 35 N with the flow field. The sample is placed into the flow field and mounted on an XYZ translation stage. Electrochemical measurements were performed using a hydrogen reference electrode (Gaskatel Germany) and an MMO iridium oxide rod as a counter electrode (CE) (METAKEM). All experiments were conducted in 2 M HClO_4_ except for the dissolution studies, in which 0.1 M HClO_4_ was used. For the latter, the S-GDE was linked to mass spectrometry: The electrolyte outlet was coupled to an online ICP-MS with a Nexion 350X interment (PerkinElmer), which was used to perform in situ stability measurements of GDEs. For the carbon corrosion studies, a HiQuad QMA 410 Quadrupol (Pfeiffer Vacuum GmbH) was connected to the gas outlet of the flow field. The flow rate was set to 5.52 ± 0.6 μL s^–1^ for the ICP-MS measurements and 2.83 ± 0.03 μL s^–1^ for the electrochemical and OLEMS experiments.

A more detailed description of the experimental procedures can be found in the Supporting Information in chapter “4. Detailed Experimental”.

## 3 Results

### 3.1 Cell Design Improvements

Typically, measurements in AMS half-cells such as the RDE and the SFC are limited to low current densities of about 5–10 mA cm^–2^ due to mass transport limitations.^7^ To facilitate the sequential approach of catalyst testing aimed at high-throughput experimentation combined with the option to integrate different mass spectrometry techniques, the SFC was chosen as a base design for the S-GDE. To implement high current density measurements on GDEs, the SFC had to be adjusted in four main areas: (1) to ensure that gaseous reactant reaches the catalyst layer effectively, a flow field was added to the setup. Furthermore, (2) the *iR* drop was decreased for simpler compensation and to remain within the compliance voltage limits of the potentiostat,^55^ (3) the CE was improved to not limit the reaction on the high surface WE, and (4) oxygen diffusion from the environment to the WE was minimized.

**Figure 1:**
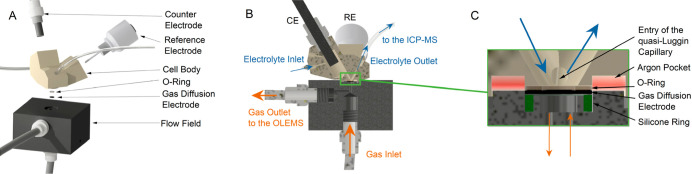
Design of the S-GDE. (A) Parts of the S-GDE represented as an explosion drawing. (B) Half section of the cell and flow field to visualize the electrolyte flow and gases management (C) close-up of the working electrode. The argon pocket (red) and the silicone ring (green) have been colored for a clearer distinction.

The resulting design of the S-GDE can be seen in Figure 1A. The cell body is made from polyether ether ketone (PEEK) to ensure chemical resistance against higher concentrated electrolytes. Both electrodes, RE and WE, can be screwed into the cell. The electrolyte can be analyzed by connecting the cell to an ICP-MS, while gaseous products can be tracked with the OLEMS (Figure 1B).

The cell body is placed on a carbon flow field. Low pressure drops and uniform concentration are desirable across the catalyst layer, which should also be achieved by the presented system.^56^ Due to the small size of the catalyst sample, the gas is only distributed by a small chamber below and not by a channel system as in other cell designs. The flow was simulated to examine the pressure distribution within the small chamber. As evident in Figure S1, the pressure is assumed to be constant across the sample. Even within the whole flow field, the pressure changes are neglectable (compare Figure S2). Like the liquid outlet, the gas outlet can be connected to external analytics, such as an OLEMS (see below). The gas stream is controlled by a flow system, with the option to use several different gases, like argon and oxygen.

A compact, circular catalyst layer can be positioned within a suitable recess in the flow field, and the cell body, featuring a standard SFC opening with a sealing ring made from POFLON, is carefully pressed onto it. A silicone ring has been inserted into the flow field below the catalyst layer to ensure airtightness.

**Figure 2:**
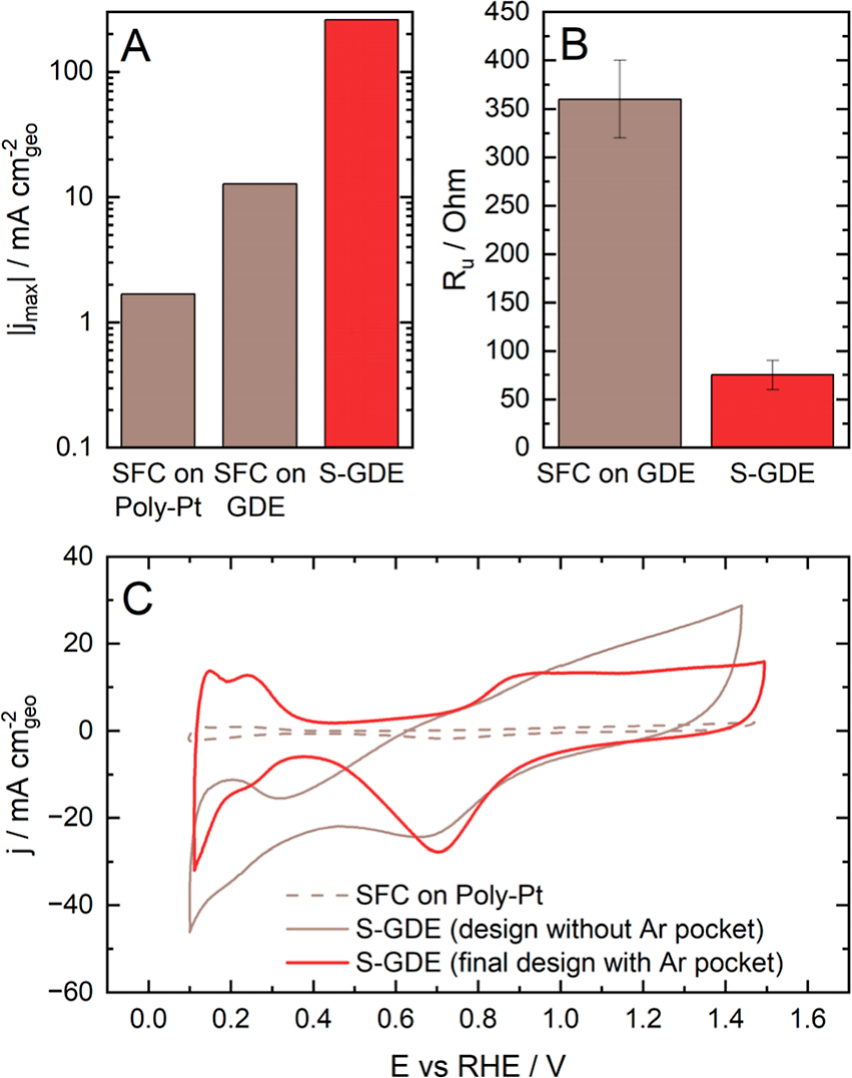
Improvements of the S-GDE in comparison to the standard SFC. (A) Measurement of the highest current density of an SFC on a Poly-Pt foil with electrolyte purged with oxygen (data extracted from^10^), a standard SFC and the S-GDE on a GDE catalyst layer and flow field with oxygen flow. (B) Uncompensated resistance of the SFC and the S-GDE in comparison. (C) Cyclic voltammetry (200 mV s^–1^) recorded in an SFC on Poly-Pt foil, a previous design of the S-GDE without an argon pocket and S-GDE as its final design, and on Pt/C GDEs (HiSPEC 4000 on Freudenberg H23C8) with a loading of 0.12 mg cm^–2^. All measurements were conducted in 0.1 M HClO_4_.

These adaptions of the S-GDE led to significant improvements. Starting with the process shown in Figure 2A, by purging the electrolyte with oxygen to screen ORR activity in the conventional SFC, a current density of approximately 1 to 2 mA cm^–2^ is reached.^10^

When the catalyst is changed from a Pt polycrystal to a GDE on a flow field with an oxygen flow, as mentioned in (1), the current density increases by 10-fold (Figure 2A, second bar). Two factors can explain this surge: first, using a more dispersive catalyst can lead to higher current densities. However, mass transport within pure AMS cells is still hindering the increase in current: Even in RDE cells, the maximal current density at the typically used rotation rates of 1600 rpm is about 6 mA cm^–2^.^57−59^ Although the SFC lowers mass transport resistance via flow, it is less effective than the transport in the RDE. Therefore, the current increase is also attributed to the enhanced oxygen availability resulting from the flow field delivering gas to the catalyst layer.

The uncompensated resistance had to be minimized to achieve the second current increase indicated in Figure 2A. The cell design was changed to reduce the *iR* drop, discussed prior as (2): the position of the RE and its channel to the catalyst layer was optimized as those primarily affect the *iR* drop.^55^ The RE was placed inside the cell, and the channel diameter connecting the flow channels to the RE chamber acting as a quasi-Luggin capillary (compare Figure 1C) was increased from 0.5 to 1 mm. The most significant impact on the resistance is the distance from the entry of the quasi-Luggin capillary to the WE.^60^ This distance is mainly influenced by the thickness of the O-ring attached to the cell (Figure 1C), so a thin ring is recommended. The O-ring of the conventional SFC has a thickness of 100 μm,^17^ yet due to the higher force utilized in this setup (35 N for the S-GDE compared to 1 N for the standard SFC^17^), a sturdier and hence thicker ring PTFE Ring was chosen. This PTFE ring is cut from a sheet of POFLON with a thickness of 250 μm, which uses silicone glue as an adhesive and compromises between robustness and slimness.

Second, the position and material of the CE were improved, as described in (3). In contrast to a conventional SFC, the design was adjusted to fit the CE inside the cell. It was previously discussed in the literature that a minimal distance between the RE and CE lowers the compliance voltage, which needs to be applied by the potentiostat.^55^ Hence, a higher current density could be reached within the boundaries of the potentiostat. High carbon corrosion is induced on the glassy carbon rod typically used as CE in SFC applications due to the occurrence of the OER as a counter-reaction to the ORR. To mitigate this effect, the glassy carbon rod was replaced by a titanium rod covered with iridium mixed oxide (Metakem, Germany). This counter electrode proved to be stable over multiple applications.

These improvements have decreased the uncompensated resistance from 360 ± 20 Ω in the standard SFC to approximately 75 ± 10 Ω in S-GDE, as presented in Figure 2B. Adjusting the RE and WE position increased the total current by 20-fold, from 12 mA cm^–2^ for the SFC on a GDE to 260 mA cm^–2^ in 0.1 M HClO_4_, the second current density increase indicated in Figure 2A.

Lastly, the WE was enclosed in an argon ring, protecting it from environmental oxygen [point (4)] by a steady gas flow, as illustrated in Figure 1C. Since PTFE has sealing characteristics, the O-ring on the cell was made from this material. A sealing ring made from silicon is integrated into the flow field. After implementing these optimizations, CVs in an inert atmosphere could be measured without the undesired effects of O_2_ leaking into the cell (visualized by comparing the two CVs measured with different cell designs of the S-GDE in Figure 2C). As the Poly-Pt, usually used as a model system for SFC applications for reference, lacks porous support, the ESCA is significantly smaller, and hence, the currents achieved in the CVs are lower.

A detailed technical drawing of the cell, including all dimensions, can be found in the Supporting Information (Figures S3–S5).

### 3.2 Electrochemical Benchmarking

In a previous publication,^45^ four different gas diffusion half-cells, which vastly differ in their cell design, were compared: (1) a large GDE half-cell setup at the Helmholtz Institute Erlangen-Nürnberg (GDE-L) with a geometrical area of 2.01 cm^2^,^54^ (2) a medium-sized commercial GDE half-cell from Gaskatel, which was customized at TU Darmstadt (GDE-M, geometrical area: 0.387 cm^2^), (3) a small GDE half-cell which is utilized at the University of Copenhagen (GDE-S, geometrical area: 0.0707 cm^2^),^37^ and (4) a modified floating electrode (MFE, geometrical area: 0.0316 cm^2^) designed at National Institute of Chemistry in Ljubljana.^61^ Despite the constructional differences between the cells, comparable data were compiled, and a best-practice approach for these cells was established. After validating the improved electrochemical performance in the previous section, the S-GDE will be compared to the established best practices in the literature. First, the electrochemical data will be discussed, followed by an examination of an adapted protocol for the smaller cell.

**Figure 3:**
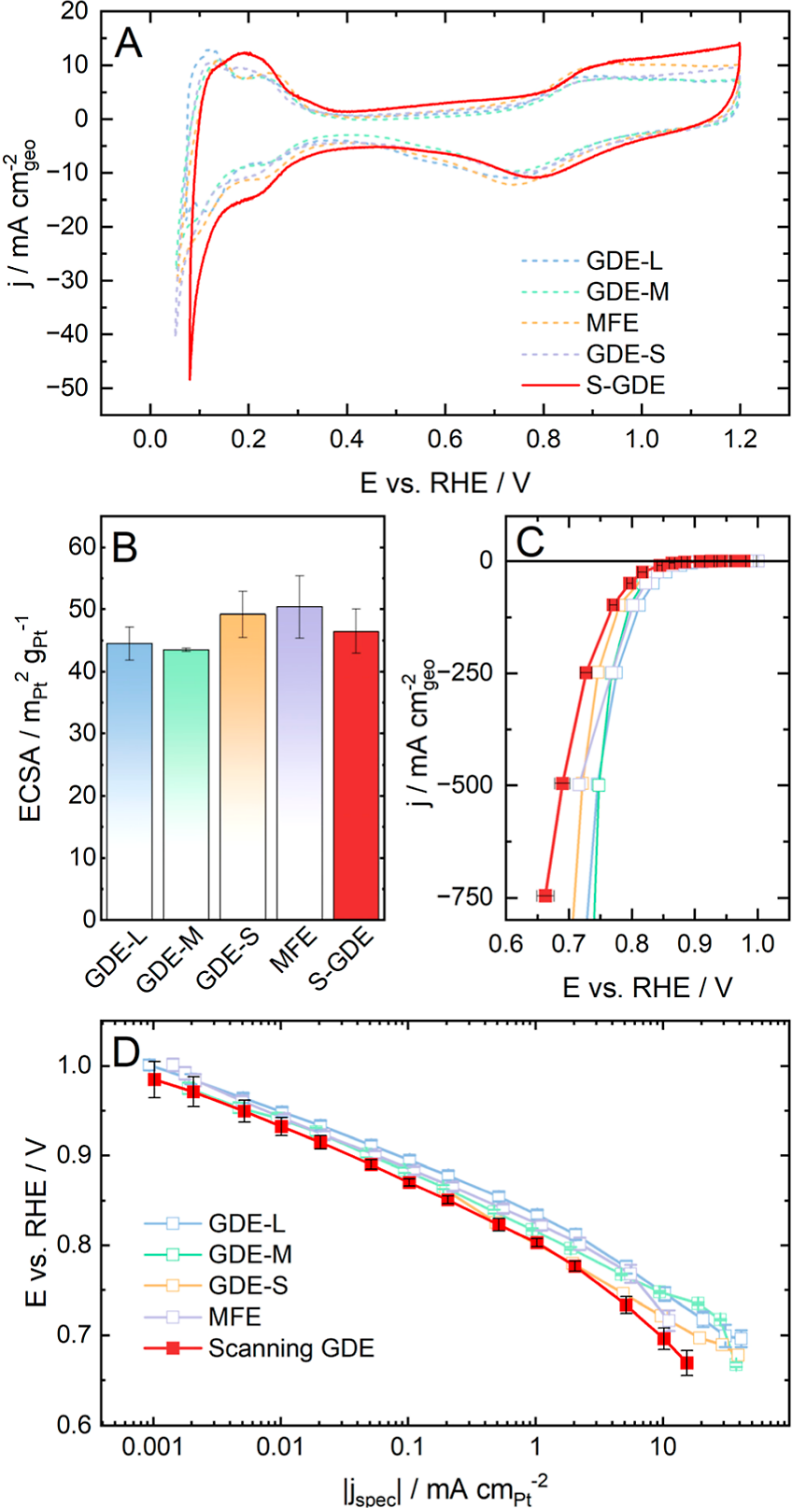
Comparison of the S-GDE to other GDE half-cells. All S-GDE & GDE-S measurements were performed in 2 M HClO_4_, and other measurements were performed in 1 M HClO_4_. A Pt/C catalyst (HiSPEC4000, 40 wt % Pt on Vulcan) with a loading of 0.1 mg cm^–2^ was used for all measurements (A) CVs from 0.05 V (0.08 V vs RHE for the S-GDE) to 1.2 V vs RHE with a scan rate of 200 mV s^–1^. (B) The ECSA was determined via integration of the H_upt_-area. (C) Polarization curves in O_2_. (D) Tafel slope of the different setups. Data, besides S-GDE, from.^45^

In line with part of the protocol established by Ehelebe et al.,^45^ (based on Pinaud et al.^36^), a cleaning step in an inert environment by either choosing Nitrogen or Argon as gas was performed (CVs from 0.08 V vs RHE to 1.2 V vs RHE). After that, CVs with a scan rate of 50, 100, and 200 mV s^–1^ were performed to determine the catalyst ECSA. As illustrated in Figure 3A, the CV resembles the ones recorded in the other different setups. Looking at the hydrogen adsorption and desorption region, the peaks are slightly shifted to a more positive potential, most probably explained by the argon flow from the back.^62^ Furthermore, the CV recorded with the S-GDE seems slightly tilted, which can indicate oxygen impurities. Nevertheless, the impact of oxygen is negligible thanks to a strong argon flow of 400 mL min^–1^ instead of the recommended 160 mL min^–1^,^45^ and the CV is similar to the others measured in the other systems overall.

As depicted in Figure 3B, the ECSA determined from CVs with a scan rate of 200 mV s^–1^ in 2 M perchloric acid is 46.5 ± 3.5 m_Pt_^2^ g_Pt_^–1^, lying in the expected range compared to the ECSA measured in the other GDE half-cell setups despite the influence of the stronger Argon flow.

Emphasis must be directed toward analyzing the polarization curve measured with the S-GDE (Figure 3C). Due to the limitations of the small cell, such as bubble formation on the CE, which can detach and block channels, reaching higher current densities is difficult. When looking at the Tafel plot (Figure 3D) for the lower current density area, the S-GDE aligns with the data recorded in the other setups. The slope is with ca. 61 mV dec^–1^ comparable to the other cells, which were in the range of 54–58 mV dec^–1^ and close to values published in the literature, reporting values from 60 to 80 mV dec^–1^ for Pt.^63^

Shifting the focus to the higher current density area, a voltage loss of the polarization curve compared to the other half-cell setups becomes apparent. Multiple factors could play into it:

(A) As previously discussed in the literature, small geometrical areas in cells lead to an overall more pronounced influence of inhomogeneities, as well as the performance and reproducibility of the data.^45^ More activation is needed to access the whole catalyst fully (see Figures S7 and S9) compared to the cells used in the presented literature. Assuming the catalyst layer is fully activated, one can see a trend correlating voltage losses and the area of the catalyst layer. The GDE-L with a geometrical area of 2.01 cm^2^ and the GDE-M with an area of 0.385 cm^2^ perform the best, while the S-GDE (0.0314 cm^2^) and GDE-S (0.0707 cm^2^) show increasing voltage losses for the higher current density region as the geometrical area shrinks due to the rising impact of inhomogeneities when the geometrical area decreases.
(B) Furthermore, due to the electrolyte’s presence, the degree of flooding in the catalyst can vary between the different setups and the pressure on the catalyst can differ in between setups, affecting the wetting behavior of the electrolyte. As the S-GDE is pressed with 35N on the catalyst layer to seal it properly, water is more likely to penetrate the pores than the other half-cells. A higher electrolyte content in the pores in the catalyst can inhibit oxygen transport to the catalyst in the microporous layer and worsen the performance.^64^

A polarization curve without electrolyte flow was recorded to exclude the electrolyte flow as a reason for the voltage loss in the S-GDE, leading to even worse performance as the bubbles from the CE were not effectively removed (see Figure S8). It can be assumed that the voltage loss can be assigned to the larger influence of inhomogeneities due to a smaller geometrical area and the presence of electrolyte in the catalyst layer. However, in summary, the S-GDE proves to have the capacity to investigate the performance of catalyst layers comparable to the other GDE half-cells in the literature.

#### 3.2.1 Adaptation of Electrochemical Protocol for the S-GDE

As mentioned previously, an adaption of the established electrochemical protocol was needed. Because the cell only utilizes a small geometrical area due to its compact design, the reasons discussed in paragraph (A) play a more significant role than in other cell designs, necessitating a better activation procedure.^54^ Hence, the electrochemical activation of the sample was increased from 20 to 50 to 150–200 CVs until no changes were observed across 10–20 CVs, which was also suggested by Pinaud et al.^36^ The effects can be studied in Figure S7 comparing different CV cycles. After 200 cycles, the first notions of activation losses became apparent. Previously, no gas flow was suggested during ECSA determination,^45^ which proved to be challenging for the setup presented. An Argon flow of 400 mL min^–1^ helped in achieving the best results. As a high flow rate can lead to more hydrogen production due to a shift of the HER onset potential, the lower potential limit was increased from 0.05 V vs RHE, as previously suggested^36,45^ to 0.08 V vs RHE. This shift had little effect on the HUP_D_ region; hence, the ECSA can still be reliably determined. Different approaches have been proposed to assess a catalyst’s activity in a GDE half-cell setup. Schmitt et al.^65^ pointed out that a standard LSV instead of current steps and subsequent electrochemical impedance measurements could be sufficient, as there are only slight changes in resistance throughout the polarization curve. Nevertheless, as the shift in potential does not depend on the current density but on the overall current in the system, a slight change in current can lead to a significant change in potential, especially when using a sizable geometrical area.^36^ The method of current steps suggested by Pinaud et al.^36^ was kept to facilitate the comparability between setups and some transferability to MEA setups. Special attention was also given to the duration of the single current steps: on the one hand, a stable potential must be reached, which can take several seconds. On the other hand, a long hold at a high current density can lead to increased bubble formation and a local shift in temperature, as discussed in other publications.^36,65^ Due to the electrolyte flow present in the cells, local changes in resistance as an effect of temperature increase can be assumed to be neglectable. Nonetheless, bubble formation is a factor to consider in small flow cells. Hence, shorter periods of constant current were introduced, and the impedance measurement interval was minimized to decrease the hold time further. Lastly, short breaks were implemented before every sequence to flush the cell with air by adding a three-way valve, resulting in effective bubble removal and reliable measurements.

A summary of the adapted protocol for the S-GDE can be found in Table S1.

### 3.3 Coupling the S-GDE to Mass Spectrometry

Besides the potential of S-GDE to be used in high throughput automated and even autonomous experiments,^10,66^ the other primary purpose of the S-GDE is its fast and reliable cell coupling to mass spectrometry, similar to the SFC.^67,68^ After establishing the electrochemical performance of the S-GDE in comparison to previously published best practices, a Pt dissolution study conducted with an ICP-MS and an investigation of carbon corrosion assessed with an OLEMS have been chosen as model systems.

#### 3.3.1 Tracking Catalyst Dissolution Online by Coupling the S-GDE to ICP-MS

While there is vast, comprehensive literature on the dissolution of Pt,^69^ discussions regarding this topic in real catalyst layers at relevant current densities are relatively sparse. Previously, the GDE-L was connected to the ICP-MS to study the effect of loading and membranes on Pt dissolution in an argon environment.^22^ Ku et al.^47^ used this approach to investigate the dissolution mechanism of the iron species within the FeNC catalyst in an alkaline environment with high current densities. Comparing two different FeNC variants, they studied the impact of oxygen flow and argon flow on iron dissolution. Surprisingly, the dissolved amount of iron was notably higher in an oxygen environment and proportionally correlated to the electrical charge.

Considering Pt dissolution in an oxygen environment, the literature is ambiguous. In general, it is assumed that the presence of oxygen can enhance Pt dissolution,^70,71^ yet there is also contradictory literature that claims that oxygen does not affect the dissolution of Pt.^72^ It has been hypothesized that OH^–^-radicals, which can form during ORR,^73^ can enhance Pt dissolution even at low potentials,^74,75^ especially in mild conditions or organic electrolytes. Noël et al.^75^ found no evidence for this in acidic electrolyte when performing ORR, while others^30^ stated that peroxide (and hence OH-radicals) can enhance the process, also in an acidic regime. Furthermore, it was suggested by Kongkanand and Ziegelbauer^76^ that an oxygen-rich environment can induce the place-exchange mechanism on dispersed nanoparticles, which is assumed to be correlated with dissolution, as early as 0.75 V vs RHE compared to 1.1 V vs RHE when oxygen is absent. However, all data in the literature presented were limited by the oxygen solubility in the electrolyte as AMS cells were used, so a high current density could not be achieved.

As the impact of gaseous oxygen on Pt dissolution in GDEs was not addressed previously, this will be explored in the current section. Before delving into new territory, the configuration consisting of S-GDE coupled with the ICP-MS will be compared against the already published GDE-L-ICP-MS setup. The protocol consists of fast and slow potential cycling, contrasting different stresses on the catalyst. It was first applied in the GDE half-cell in an argon environment, followed by its implementation in the S-GDE in an argon and oxygen environment. The loading and the electrolyte were kept constant.

**Figure 4:**
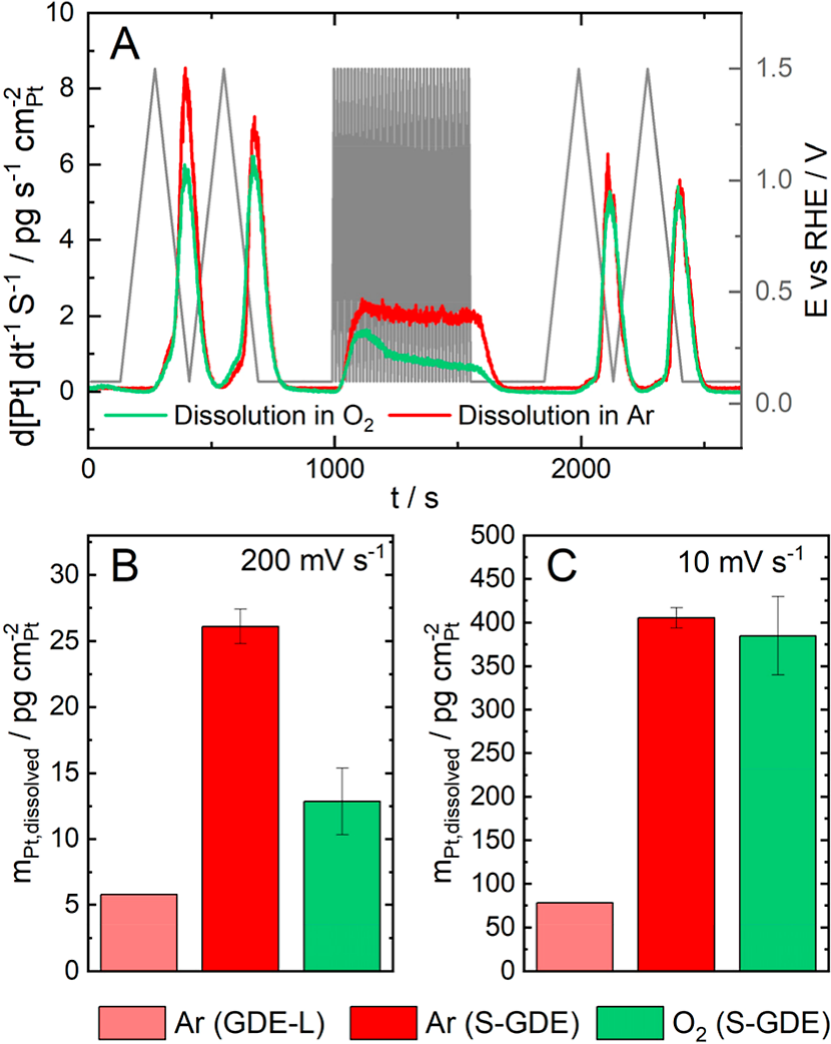
Pt dissolution data measured via the S-GDE and GDE half-cell setup coupled to the inductive couple plasma mass spectrometer in 0.1 M HClO_4_. (A) Pt dissolution profiles from Pt/C GDEs (HiSPEC 4000 on Freudenberg H23C8) with a loading of 0.12 mg cm^–2^ in Ar (red) and O_2_ (green) measured in the S-GDE. (B,C) Quantitative Pt dissolution during the fast cycling (B) and slow cycling at the end (C) normalized by CV in Ar (GDE-L and S-GDE) and O_2_ (S-GDE).

Employing oxygen during the potential cycle led to increased ORR activities with high current densities of up to −257 mA cm^–2^, as depicted in Figures S10 and S11. Comparing the integrated amount of Pt during the protocol in an argon environment recorded in S-GDE and GDE-L (visible in Figure 4B,C) reveals a higher dissolution in the S-GDE as opposed to the GDE-L. 5.8 pg cm_Pt_^–2^ dissolved in the GDE-L during one fast cycle (200 mV s^–1^) and 78.1 pg cm_Pt_^–2^ during one slow cycle (10 mV s^–1^), and 26.1 pg cm_Pt_^–2^ and 405.4 pg cm_Pt_^–2^ dissolved in the S-GDE, respectively. Employing a different system can independently impact results, irrespective of the catalyst. It can be hypothesized that the flow of the electrolyte can affect the amount of detected Pt, inhibiting the repositioning of the catalyst by transporting the catalyst to the mass spectrometer and other mixing effects. However, given the microporous structure of the catalyst layer, most of the redepositing effects will occur in small pores, mostly unaffected by the flow in the bulk electrolyte. Yet, as the GDE-L is a closed system, products accumulate in the electrolyte. Although it can be accounted for, this preconcentration may still obscure the dissolution behavior, as the dissolution may depend on the concentration of dissolved species considering the Nernst equation. Furthermore, a collection efficiency factor must be determined when using the GDE-L for online dissolution studies, as only part of this electrolyte is analyzed in the ICP-MS (compare section “4.3 GDE-L-ICP-MS measurements” in Supporting Information). While this approach proves to be reliable, it can be argued that analyzing all the electrolyte introduces less error. As already discussed, the last factor is also reflected by the voltage loss visible in the polarization curves in Figure 3: it hints at a higher level of flooding in the catalyst system, which could affect the transport of Pt within the catalyst layer. Ehelebe et al.^22^ discussed the effect of different transport media within the catalyst layer on the amount of dissolved Pt, leading to a higher dissolution in a completely flooded environment than in a less flooded environment. Nevertheless, contrasting the amount of dissolution to the results obtained with the SFC, the dissolved amount is significantly lower, which becomes apparent in Figure S12, comparing the dissolved amount of Pt per potential cycle as a function of loading in different systems. Hence, one can argue that the discrepancy in the amount of dissolved catalyst can be due to differences, while the level of flooding is comparable. It is also likely that the dissolution measured with the S-GDE is more representative, as no collection efficiency has to be calculated nor does the Pt accumulate in the electrolyte. On the other hand, AMS cells, such as the SFC, are entirely flooded, leading to a meaningful difference in the dissolved amount of catalyst compared to the presented GDE half-cell setups. Ensuring no effect of the RHE employing Pt as part of the electrode, a dissolution study on mere carbon was conducted, and no significant dissolution was recorded, as evident in Figure S9.

Despite the different cell designs, the trends are clearly comparable. The time-dependent Pt dissolution graph recorded with the GDE-L can be found in Figure S6. In both systems, the Pt dissolution is higher during the first CVs due to the initial dissolution of low coordination sites typical on fresh samples.^77,78^ In addition, the typical two peaks observed during slow potential cycling on a Pt catalyst are apparent: a small dissolution peak can be observed during the positive scan, followed by a significantly increased dissolution peak during the negative scan period. As already investigated in previous dissolution studies, the small dissolution peak can be ascribed to anodic dissolution happening while Pt oxide is formed. When the Pt oxide is reduced during the anodic scan, a significantly higher amount of Pt dissolves.^69,79^Figure 4B,C illustrates that the dissolved amount also depends on the scan rate, which aligns with the established literature.^80^

Examining the dissolution curve in the presence of oxygen reveals a different activation process of the catalyst compared to an argon environment during the fast cycling: A decrease in dissolution over time becomes apparent, stabilizing after a period, while the Pt dissolution in an Ar environment remains constant over the period of the fast potential cycling. Oxygen could affect the first dissolution of low-coordinated sites, pointing to a different dissolution mechanism, as previously considered by Matsumoto et al.^71^

Additionally, a correlation between the impact of the gas and the difference in the dissolved amount seems to depend on the scan rate (see Figure 4B,C). At fast scan rates, the Pt dissolution seems lower in an oxygen environment compared to an argon environment. Lowering the scan rate, however, leads to comparable dissolved quantities. Yet, the overall trend of the Pt dissolution seems to correspond to the dissolution in an argon environment: During slow scans, the anodic and cathodic dissolution peaks become visible and slower scan rates lead to more dissolution per CV.^22^

In literature, different mechanisms have been proposed on how oxygen can enhance dissolution,^81^ especially in low voltage window,^71,75,76^ yet no clear trend has been established so far.^72,75^ Different factors are discussed which could contribute to the absolute amount of oxygen, such as partial pressure^81^ and form of the electrode.^71^

Investigations on single-crystal surfaces have shown increased reactivity of Pt sides closed to O_2_-occupied Pt sides.^82^ Furthermore, it was revealed that oxygen led to enhanced roughening on the surface, while potential oxygen contrasted to its absence, as low as 0.6 V vs RHE.^71^ This suggests a place-exchange reaction taking place, which was later confirmed.^76^ The place-exchange reaction will likely influence the dissolution of Pt sides at defects or smaller particles, which can explain the previous activation mechanisms.

Yet, to deepen the understanding of the Pt dissolution process on a fundamental level, more Pt single crystal dissolution studies in the presence of oxygen must be carried out, as already conducted for inert environments.^83,84^

All the discussed literature was carried out in AMS half-cells. There are suggestions that elevated oxygen pressure conditions can lead to greater degradation^85^ due to the impact of oxygen.^86^ The effect of oxygen flow on Pt dissolution in a real catalyst layer is not widely discussed yet. It can be postulated that different processes might take place, and the dissolution mechanism is governed by different factors in catalyst layers employed in fuel cells than in the previously described model systems.

It can be concluded that the mechanism of Pt dissolution in an oxygen environment is not clearly understood yet. The mechanism of how oxygen can affect dissolution remains ambiguous due to the lack of literature on the correlation between Pt oxide formation and the presence of oxygen. Furthermore, it can be postulated that the place exchange of oxygen in an O_2_ can affect the surface composition and enhance the dissolution, yet closer studies investigating the impact of all the previously mentioned factors are needed.

Overall, these results indicate the strengths of the presented system: The presented data suggest that Pt dissolution also depends on the environment of the catalyst in the MEA, which can be more closely mirrored in the presented S-GDE than in the standard SFC.

#### 3.3.2 Monitoring the Gaseous Products in Real-Time by Coupling the S-GDE to the OLEMS

The detection of CO_2_ as a product of carbon corrosion was chosen as an exemplary model reaction to verify the utilization of an S-GDE-OLEMS system. As GDE half-cells are applied in various fields, this technique can also be employed in other electrochemical research areas, such as CO_2_ conversion.

It has been previously shown that CO_2_ is produced from carbon in an acidic environment; hence, it can be used as a metric for carbon corrosion.^87^

Carbon corrosion has been extensively studied, as the oxidation of the support and its deterioration can lead to a change in surface structure or pore distribution, decreasing the performance of the fuel cell.^88,89^ Multiple studies already investigated carbon corrosion by quantifying CO_2_.^50,90^ Thus, applying ASTs with potential cycling from 0.1 to 1.5 V vs RHE to examine the degradation of the fuel cell cathode and using a Fuji Electric ZRH IR CO_2_ gas analyzer to quantify the CO_2_, Young et al. found that the loss of carbon was independent of the ionomer content in the catalyst layer.^90^ Cremers et al., on the other hand, investigated the impact of different carbon supports on the loss of the ECSA.^50^ Both examples show the necessity of investigating carbon corrosion on fuel cell electrodes, as this can reveal the impact of the electrode structure on carbon corrosion.

With water present, carbon is thermodynamically unstable for potentials above 0.207 V vs RHE,^88^ and hence, carbon degradation can be considered at a fuel cell cathode, assuming that ORR takes place. Yet, due to the slow kinetics of the reaction, it is broadly assumed that it is neglectable below 1 V vs RHE.^29^ It is presumed that the main contributing aspect to carbon corrosion is the reverse current decay, increasing the cathode’s potential up to 1.44 V.^91^

After a cleaning cycle, as explained in Table S1, the protocol of potential steps was applied. The gas stream of argon was lowered to 50 mL min^–1^ to decrease the dilution of the product.

**Figure 5:**
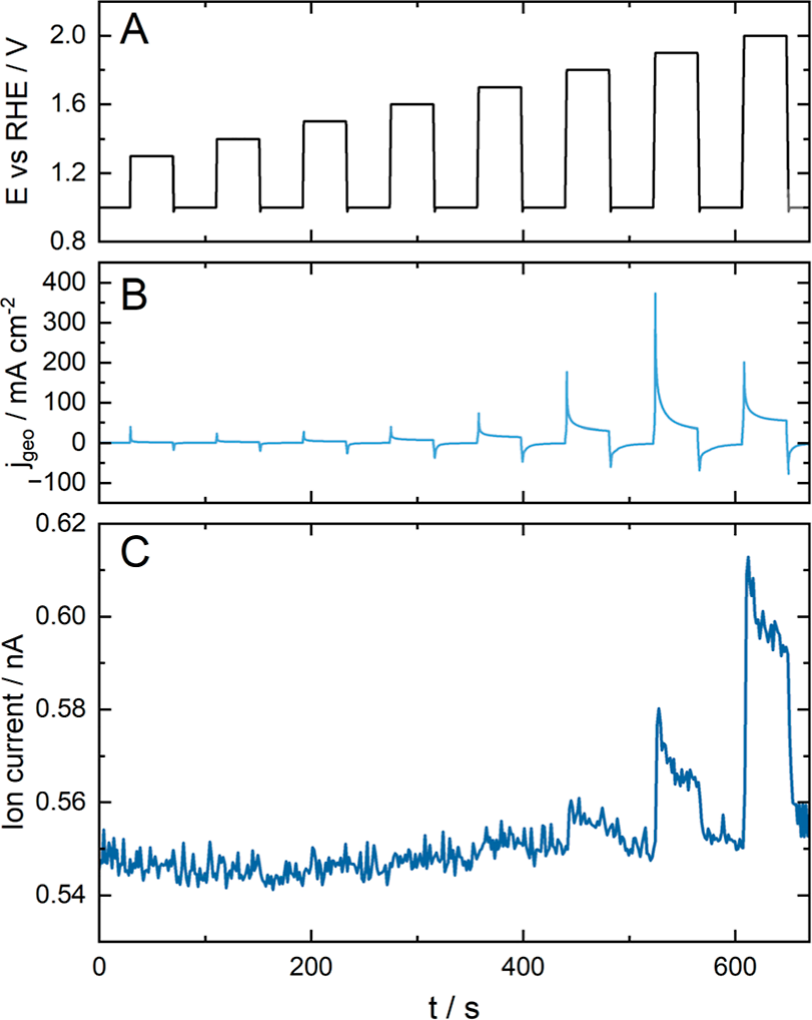
Carbon Corrosion indication by CO_2_ measured on Pt/C GDEs (HiSPEC 4000 on Freudenberg H23C8) with a loading of 0.12 mg cm^–2^ in 2 M HClO_4_ after performing cleaning cycles. (A) Applied protocol, the resulting (B) current density, and (C) ion current of CO_2_.

As the data presented in Figure 5 serve as proof of concept, the chosen protocol is aimed at inducing corrosion solely: Potential holds from 1.3 to 2 V, increasing by 0.1 V per step, was chosen as a test protocol (compare Figure 5A). To ensure regeneration of the CO_2_ baseline, a potential of 1 V vs RHE was applied between each step. Each potential was held for 40 s.

A significant increase in current during the potential steps is visible from 1.6 V vs RHE with a current density of about 7.5 mA cm^–2^, which can be mainly attributed to the oxygen evolution reaction. Thermodynamically, the reaction sets in at 1.23 V vs RHE, but due to the sluggish nature of the reaction and the consequential overpotential, especially on Pt compared to other noble metal catalysts, such as Ir,^92^ a significant current can only be observed at higher potentials. The current density increases for each potential hold until it reaches 55 mA cm^–2^ at 2 V vs RHE.

However, as the CO_2_ signal in Figure 5C indicates, a part of the current measured can be assigned to carbon corrosion. Initially, the ion current shows a baseline value of approximately 0.55 nA until a modest increase can be observed when 1.8 V vs RHE is applied. On incrementally raising the potential, the signal response amplifies correspondingly, leading the signals to 0.58 nA, stabilizing around 0.565 and 0.61 nA, and decreasing to 0.59 nA for 1.9 and 2 V, respectively. These sharp increases in the CO_2_ signal commonly occur when there is a change in potential.^93^ The decrease in the signal is mainly attributed to a built-up of a protecting passivation layer inhibiting further oxidation^93^ or catalyst layer degradation.^94^

However, considering the Vulcan catalyst, the average loading, and the acidic electrolyte employed in this study, the carbon corrosion should be easily measurable at potentials upward from 1 to 1.2 V, as this was reported previously in the literature with an experimental design comparable to that of the S-GDE.^50,95^ Since the late onset of detection of CO_2_ cannot be explained by an electrochemical perspective, the setup itself will be further examined: It can be assumed that the collection efficiency achieved by the system is too low to record CO_2_ in relevant potential windows (1–1.5 V vs RHE).^90^ No GDE-OLEMS study has been conducted which could serve as a direct comparison, although Niether et al.^49^ developed an OLEMS cell for high-temperature applications to test GDEs with gaseous educts, yet it was not optimized for high current densities. On investigating the low collection efficiency, the S-GDE will be contrasted with the different cells used in the literature. Compared to MEA cells that are used to investigate carbon corrosion, the electrode area of the fuel cell is larger than in the presented system, leading to a higher concentration of CO_2_ in the analyzed gas stream.^50^ This also applies to the cell, as mentioned earlier by Niether et al.^49^ Furthermore, a smaller and more consistent sampling channel within Niether et al.’s^49^ setup leads to less dilution caused by turbulence in the system. The previously discussed SFC was employed within an OLEMS system to study carbon corrosion.^95^ The geometrical area of the working electrode resembles the S-GDE’s area, yet the gaseous products were sampled directly above the working electrode; hence, no additional gas stream could decrease the product’s concentration.

Nonetheless, it has been proved that the S-GDE can be utilized with an OLEMS. Improvements must be made to improve the collection efficiency: The inlet of the mass spectrometer can be placed closer to the working electrode as in the standard SFC. Furthermore, the argon flow has to be optimized to increase the product concentration. Lastly, the area of the catalyst can be increased to raise the amount of CO_2_ being produced.

## 4 Conclusions and Outlook

The S-GDE provides a powerful tool to enhance the throughput of fundamental studies in electrocatalysis, considering the effects of real catalyst layers’ characteristics on the stability and performance of the electrode. The presented cell can streamline analyzing the various variables influencing the electrode’s stability and performance. Furthermore, high current densities can be achieved compared to the conventional SFC.

The cell was adapted for high current density application using the conventional SFC as a base design. A flow field was constructed for the cell, and the WE and RE were adjusted, which led to an increase of the maximal current from 2 mA cm^–2^ in the conventional SFC to 240 mA cm^–2^, and the resistance was decreased from 360 ± 20 Ω in the conventional SFC to 75 ± 10 Ω in 0.1 M HClO_4_.

The new cell has been electrochemically validated by benchmarking it against other cells. The electrochemical results were in agreement with the published systems. Due to the small design of the cell, the protocol had to be optimized for the compact S-GDE. As a result, a remarkable current density of 750 mA cm^–2^ can reliably reach 0.66 V vs RHE. Hence, the cell can be a viable tool to identify trends for different catalysts.

Connecting the cell to in-operando mass spectrometry, the Pt dissolution of real catalyst layers in an inert and oxygen environment has been studied as a prototype with an ICP-MS. Unlike that reported in the literature, the dissolution of Pt in oxygen was found to be decreased for fast potential cycling. This finding further emphasizes the importance of investigating real catalyst layers, as many variables, such as the mass transport depending on the electrode structure, cannot be mirrored in model systems. Furthermore, it was shown that carbon corrosion could be studied by analyzing the exhaust gas stream with an OLEMS.

On another note, all measurements have been carried out at room temperature. As catalyst behavior also depends on the temperature, a temperature-controlled flow field could improve the cell design. The conventional SFC can be used for automated high-throughput approaches,^66,96^ so an adaption to the S-GDE’s flow field to analyze multiple catalysts would be beneficial for accelerating catalyst layer testing aimed at high-throughput analysis.

Overall, it has been demonstrated that S-GDE works as a half-cell in a functional GDE system, achieving elevated current density while offering seamless integration with external mass spectrometry. The new cell can not only be applied to the Pt/C catalyst layer but also has the potential to be used as a tool in other fields, such as CO_2_-reduction, water electrolysis, non-noble ORR catalysts like FeNC, or the investigation of air diffusion electrodes.

## Supporting Information Available

Pressure gradient simulation, technical dimensions of the flow field and the cell, in-depth description of the experimental part, cleaning CVs, adapted protocol for the S-GDE, different factors affecting the experiments, graphs on the effect of oxygen and argon, and comparison of the dissolved Pt amount in GDE-L, SFC and S-GDE (PDF)

## Abbreviations

AMS: aqueous model system
CCM: cathodic catalyst layer
CE: counter electrode
CV: cyclic voltammetry
ECSA: electrochemical surface area
GDE: gas diffusion electrode half-cell
HOR: hydrogen oxidation reaction
ICP-MS: inductively coupled plasma-mass spectrometer
OLEMS: online electrochemical mass spectrometer
ORR: oxygen reduction reaction
PEMFC: proton exchange membrane fuel cell
Pt: platinum
RDE: rotating disk electrode
RE: reference electrode
SCEM: scanning electrochemical microscopy
SFC: scanning flow cell
S-GDE: scanning gas diffusion electrode half-cell
WE: working electrode
