# Supplementary material for: Scanning Gas Diffusion Electrode Setup for Real-Time Analysis of Catalyst Layers
Source: ACS Meas Sci Au. 2024 Jul 12;4(5):515–27. doi: 10.1021/acsmeasuresciau.4c00018 (PMC11487660; doi:10.1021/acsmeasuresciau.4c00018)
Supplement: Supplementary file 1 — tg4c00018_si_001.pdf [file tg4c00018_si_001.pdf]

Supporting Information:

Scanning Gas Diffusion Electrode Setup for Real-Time Analysis of Catalyst Layers

Ina Reichmann*[1,2], Vicent Lloret[1], Konrad Ehelebe[1,2], Pascal Lauf[1,2], Ken Jenewein[1,2] Karl J. J. Mayrhofer[1,2] and Serhiy Cherevko*[1]

[1]Forschungzentrum Jülich GmbH, Helmholtz Institute for Renewable Energy (IEK-11), Cauerstraße 1, 91058 Erlangen, Germany

[2]Friedrich-Alexander-University Erlangen, Nürnberg, 91058 Erlangen, Germany

Content

1. Pressure gradient simulations using COMSPOL Multiphysics ... 3
2. Technical Dimensions of the Flow Field ... 4
3. Technical dimensions of the cell ... 5
4. Detailed Experimental ... 6
    4.1 Preparation of the Fuel Fell Electrode ... 6
    4.2 S-GDE Setup and Control ... 6
    4.3 GDE-ICP-MS Measurements ... 6
5. Cleaning CVs ... 8
6. Adapted Protocol for the S-GDE ... 9
7. Assessing the Influence of Different Factors on the Experiment ... 9
    7.1 The Effect of Cleaning on the Catalyst and the Electrolyte Flow ... 9
    7.2 The Effect of the Reference Electrode on the Platinum Dissolution ... 10
8. Further Graphs on the Effect of Oxygen and Argon ... 11
9. Comparison of the Dissolved Pt Amount in the SFC, GDE-L and S-GDE ... 12

References ... 13

1. Pressure gradient simulations using COMSPOL Multiphysics

The simulation was executed with the help of the Element software COMSOL Multiphysics 5.5. Part of the flow field was inverted, and the catalyst layer was assumed to be not gas-permeable. A simplified technical sketch of the inverted flow field can be found in Figure S2. "Fine" was chosen as an element size for the mesh, and it was set to "physics controlled". The inlet flow was set to 0.14 m s^{-1} mimicking the oxygen volume flow, and the outlet pressure was set to atmospheric pressure. The pressure distribution was calculated via Navier-Stokes equations using a laminar flow with a compressible fluid and a temperature of 293K.

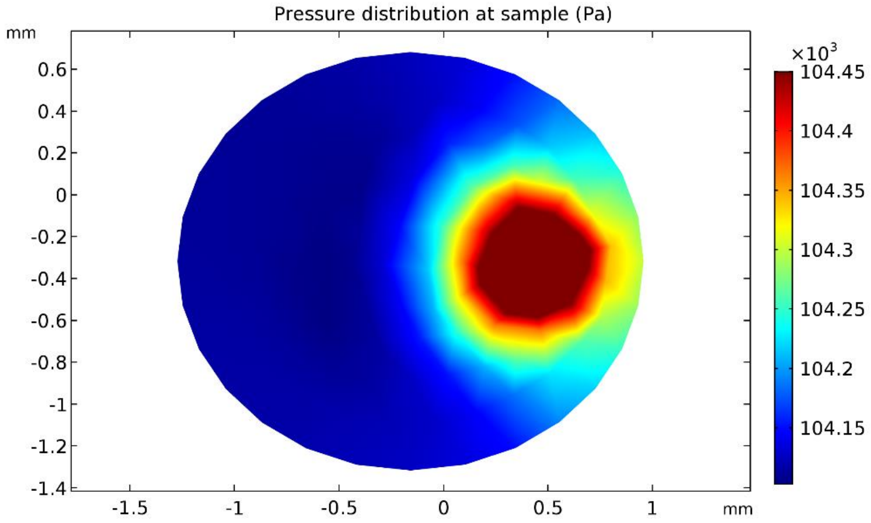

Figure S1: The simulated pressure distribution on the backside of the catalyst for oxygen.

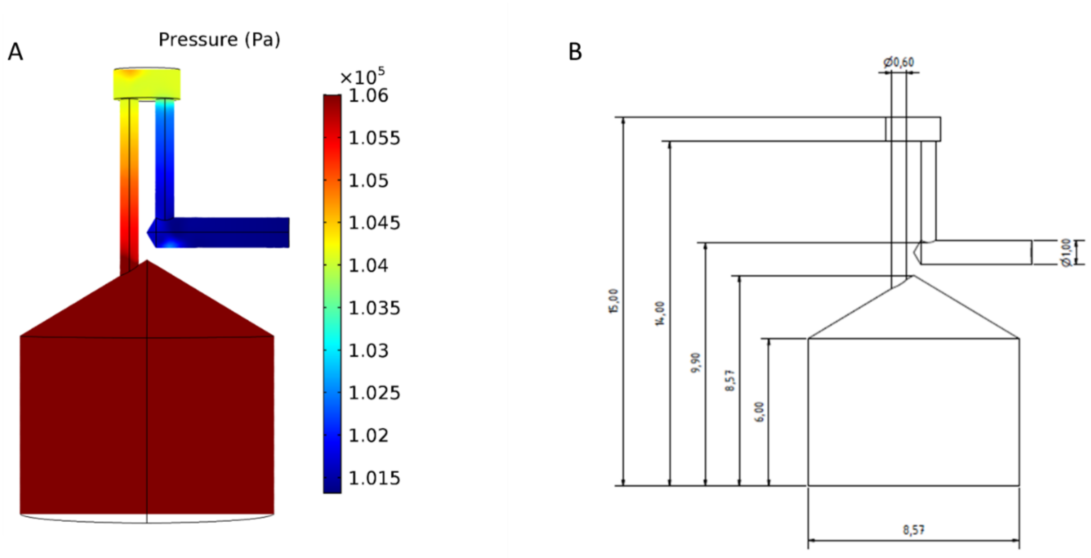

Figure S2: (A) The pressure distribution in the whole part of the flow field (B) and its corresponding dimensions in mm

2. Technical Dimensions of the Flow Field

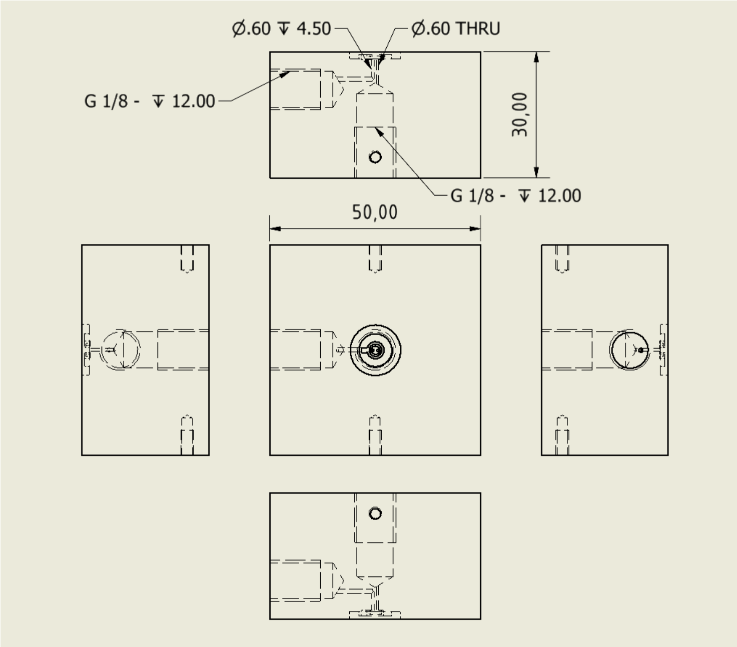

Figure S3: Dimensions of the flow field in mm

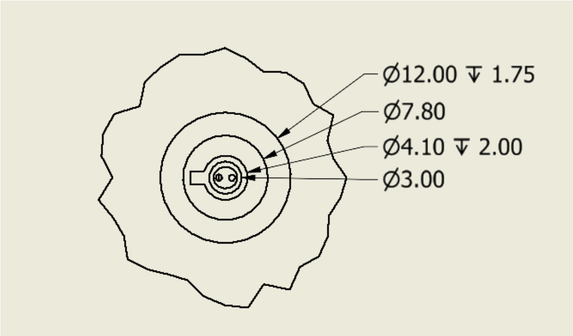

Figure S4: Zoom on the rings (dimensions in mm)

3. Technical Dimensions of the Cell

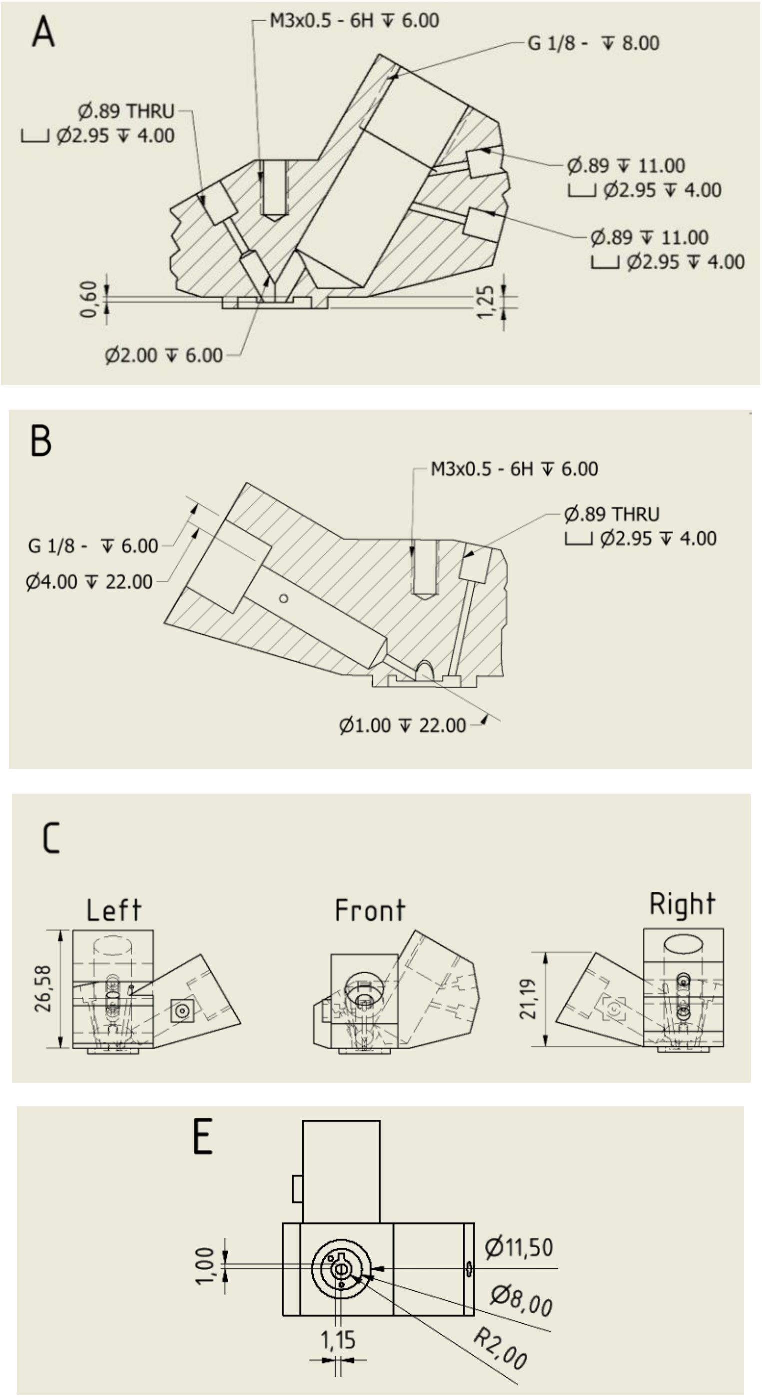

Figure S5: Dimensions of the PEEK cell, all dimensions given in mm (A) Counter electrode compartment and flow channels (B) Reference electrode compartment and inlet to the Ar pocket (C) Front and side views of the cell (E) Dimensions of the rings on the bottom

4. Detailed Experimental

4.1 Preparation of the Fuel Fell Electrode

The employed spray coater was a Multi-Axis Spraying System from Sonotek Cooperation, USA. The ink used contained 1 wt% solids in a solvent mixture of 20 wt% isopropyl alcohol (EMSURE 1-Propanol for analysis, Merck Germany) in ultrapure water (Merck Millipore, Milli-Q). The solid consisted of a 30 wt% ionomer (Nafion D520, Ion Power). To achieve good mixing, the ink was homogenized with an ultrasonic horn at 60 W for 20 min at 0°C. The ink was deposited onto a Freudenberg H23C8—gas diffusion media with the spray coater. The ink's flow rate and spray coater's speed led to a 4.78 µg ±0.13 µg cm^{-2} deposition rate per cycle. To determine the Pt loading, the samples were weighed (Sartorius Cubis, ±0.001 mg) before and after spray coating. The electrodes prepared had a loading of 0.1 mg cm^{-2} (ICP-MS) for the electrochemical evaluation and 0.12 mg cm^{-2} (OLEMS) for the mass spectrometry measurements.

Samples were cut out with a diameter of 4 mm, and the utilized geometric area was 0.0314 cm^2.

4.2 S-GDE Setup and Control

The S-GDE is mounted above the flow field with a force sensor (ME Messinstrumente GmbH, KD45 50N/VA/HT), controlling the contact pressure of 35 N with the flow field. The sample is placed into the flow field, which is mounted on an XYZ translational stage (Physik Instrumente, Germany M-403), allowing the flow field to be precisely positioned below the cell. This setup enables the cells to be quickly moved and the catalyst layer easily replaced.

Electrochemical measurements were performed with a potentiostat (BioLogic, SP 150), utilizing a custom-made Hydrogen (Mini-Hydroflex, Item number 81020, gaskatel, shaft lengths: 15 mm) Reference Electrode (RE) and an MMO rod from METAKEM as a counter electrode with a length of 7 cm. The electrical contract was established by an electrical wire connected to the flow field, the counter, and the reference electrode. The electrolyte was prepared via dilution of concentrated $HClO_4$ (VWR, Suprapure, 70-72%) with ultrapure water (Merck Millipore, Milli-Q). The electrolyte was constantly purged with argon in a separate compartment to ensure no oxygen was present in the liquid phase. Everything in the setup was controlled and synchronized via an in-house LabView software, besides the potentiostat, which utilizes EC lab.

Daily calibration was conducted for Pt with a four-point calibration curve (0, 0.5, 1, 5 µg L^{-1}), with solutions which were freshly prepared from standard solutions (Merck Centripure). Ensuring a good performance of the system, 5 µg L^{-1} of Re was used as an internal standard. The recorded response time was 40 ± 7 seconds. As soon as the cell contacts the catalyst, the time is stopped until the "contact peak" is measured by the mass spectrometer. This time is later subtracted from the data, aligning both electrochemical and ion count signal.

For the carbon corrosion studies, a HiQuad QMA 410 Quadrupol (Pfeiffer Vacuum GmbH) with two turbo pumping stations (HiCube 300 H classic and HiCube 30 Eco, also Pfeiffer Vacuum) was applied. The exhaust of the gas flow field was connected to the inlet steel capillary of the mass spectrometer.

4.3 GDE-ICP-MS Measurements

As a reference, the protocol described in the paper was performed in the GDL-L setup connected via a small capillary close to the Working Electrode to the inductively coupled plasma mass spectrometer,

which has been published prior.[1, 2] The calibration curve has been carried out as described in the in the chapter above.

As only part of the electrolyte is analyzed, the collection efficiency (CE) must be calculated to quantify the dissolution.

$$CE = \frac{m_{Pt,\ ICP-MS}}{m_{Pt,total}} = \frac{m_{Pt,ICP_{MS}}}{m_{Pt,bulk,end} - m_{Pt,bulk,start} + m_{Pt,ICP-MS}} \quad \text{S1}$$

Whereas $m_{Pt,\ ICP-MS}$ is the total amount of platinum detected by the ICP-MS and $m_{Pt,total}$ is the whole dissolved amount of the catalyst. The concentration was measured at the start ($m_{Pt,bulk,start}$) and at the end of the measurement ($m_{Pt,bulk,start}$) to calculate the amount of dissolved platinum in the bulk electrolyte. Two bulk samples were taken to assess the concentration at the end of the measurement: One right after the measurement and the second after thorough mixing. The dissolved mass has been calculated from the average of the two. The collection efficiency was calculated as 54.31%, and the flow rate was set to 0.18 ml min^{-1}. The reported response time was 5 seconds, and its determination was discussed in the previous chapter.

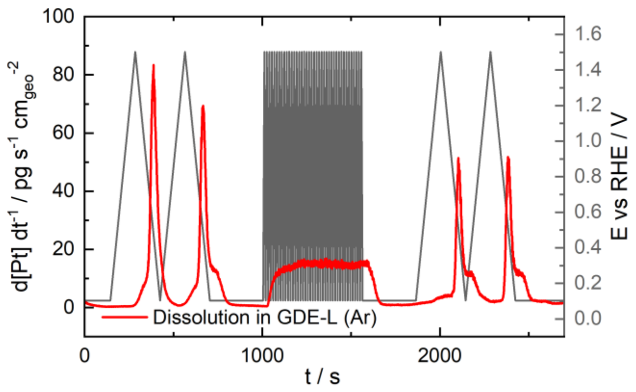

Figure S6: Pt dissolution profiles from Pt/C gas diffusion electrodes (HiSPEC 4000 on Freudenberg H23C8) with a loading of 0.12 mg cm^{-2} in Ar (GDE-L half-cell setup coupled to the Inductive Couple Plasma Mass spectrometer in 0.1 M $HClO_4$)

5. Cleaning CVs

300 cleaning CVs were performed to find an optimal required amount to obtain reliable electrode condition. The sample stabilized between cycles 100 and 200; slight activation losses could be seen for cycle 300 as the CV decreased in size.

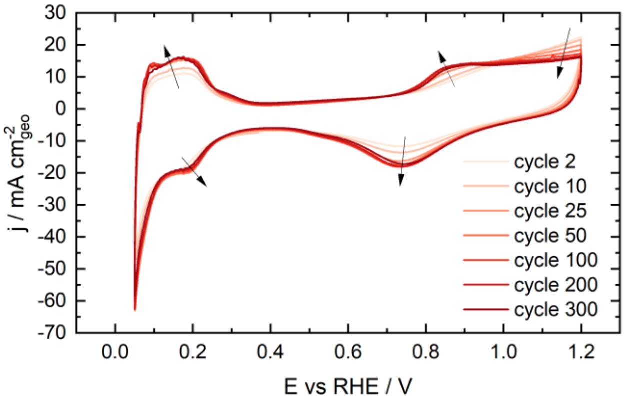

Figure S7: 300 cleaning CVs recorded with a scan rate of 200 mV s^{-1}. The flow rate of the electrolyte (2M $HClO_4$) was 2.83 ± 0.03 µL s^{-1}, and the volume flow of the argon stream was 400 ml min^{-1}. The used sample had a loading of 0.1 mg cm^{-1}

6. Adapted Protocol for the S-GDE

Table S1: Electrochemical protocol for standard analysis.

	Step	*Electrochemical Technique*	*Parameters*	
1	Electrochemical cleaning	Cyclovoltammograms (CV)	Gas purge (Flow rate)<br>Potential limits<br>Scan rate<br>Number of cycles	Ar (400 ml min^{-1}),<br>0.08 – 1.2 V vs. RHE<br>200 mV s^{-1}<br>~ 100-150 (until CV is stable)
2	ECSA determination (H_{upd})	CV	Gas purge (Flow rate)<br>Potential limits<br>Scan rate<br>Number of cycles<br>iR-compensation	Ar (400 ml min^{-1}),<br>0.08 – 1.2 V vs. RHE<br>50/100/200 mV s^{-1}<br>5 each<br>95 % in-situ correction + 5 % post-corr.
3	Oxide reduction	Chronoamperometry (CA)	Gas purge (Flow rate)<br>Potential<br>Time	Ar (400 ml min^{-1}),<br>0.10 V vs. RHE<br>5 min
4	Polarization curve (O_2)	Open circuit potential (OCP)	Gas purge (Flow rate)<br>Time	O_2 (200 ml min^{-1}),<br>10 min
		Galvanostatic steps coupled with impedance spectroscopy (GEIS)	Gas purge (Flow rate)<br>Current steps (hold time)<br><br><br><br><br><br><br><br><br>EIS frequency range<br>EIS amplitude<br>iR-compensation	O_2 (200 ml min^{-1})<br>- 0.05/-0.10 mA<br>/-0.25/-0.5/-1/-2.5 mA, -5/-10 mA cm^{-2}<br>(20 s),<br>pause to flush with air<br>-25/-50/-100/-250 mA cm^{-2} (15s)<br>pause to flush with air<br>-0.5/-0.75A cm^{-2} (5s)<br>pause to flush with air before backward<br>Scan<br>f= 200 kHz – 1000 Hz<br>10 % of current (min. 5 mA cm^{-2})<br>100 % post-correction

7. Assessing the Influence of Different Factors on the Experiment

7.1 The Effect of Cleaning on the Catalyst and the Electrolyte Flow

More cleaning cycles were applied to prevent performance loss from a lack of cleaning/activation. The polarization curve is still in the margin of error, and similar results were also achieved with the cleaning procedure mentioned above.

Stopping the flow decreased the performance, probably due to bubble accumulation on the catalyst layer. Hence, the electrolyte flow did not compromise the performance.

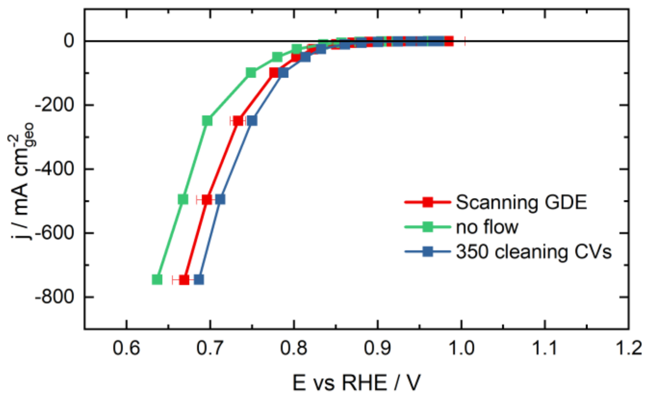

Figure S8: Polarization curves in O_2. The flow rate of the electrolyte (2 M $HClO_4$) was 2.83 ± 0.03 µL s^{-1}, and the volume flow of the argon stream was 400 ml/min. The used sample had a loading of 0.1 mg cm^{-1}.

7.2 The Effect of the Reference Electrode on the Platinum Dissolution

An experiment was performed on a GDL (Freudenberg H23C8) to ensure no leaching of Pt from the RE. The background is in the range of picograms normalized by the geometric area of the cell (0.0314 cm^2), hence neglectable.

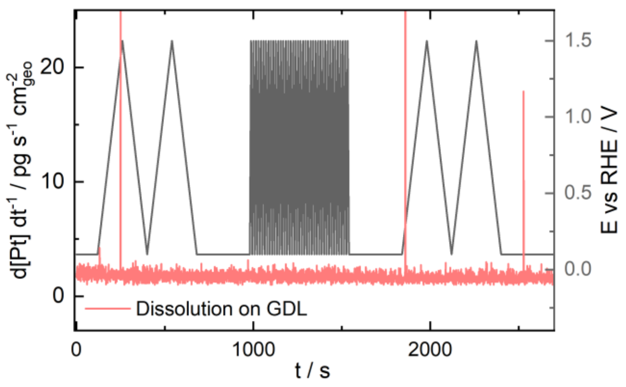

Figure S9: Pt dissolution profiles measured on Freudenberg H23C8 in Ar. (S-GDE coupled to the Inductive Couple Plasma Mass spectrometer in 0.1 M $HClO_4$.)

8. Further Graphs on the Effect of Oxygen and Argon

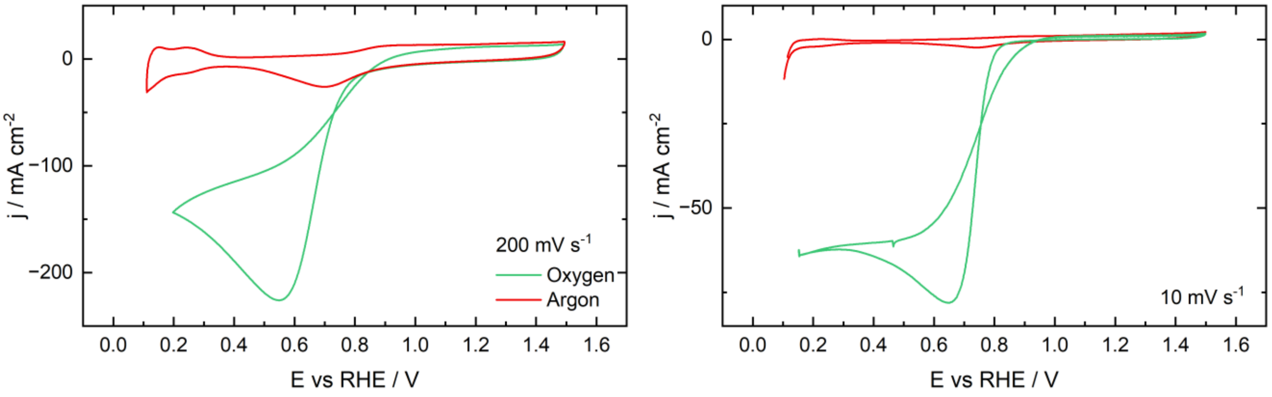

Figure S10: Cyclic voltammetry on Pt/C gas diffusion electrodes (HiSPEC 4000 on Freudenberg H23C8) with a loading of 0.12 mg cm^{-2}

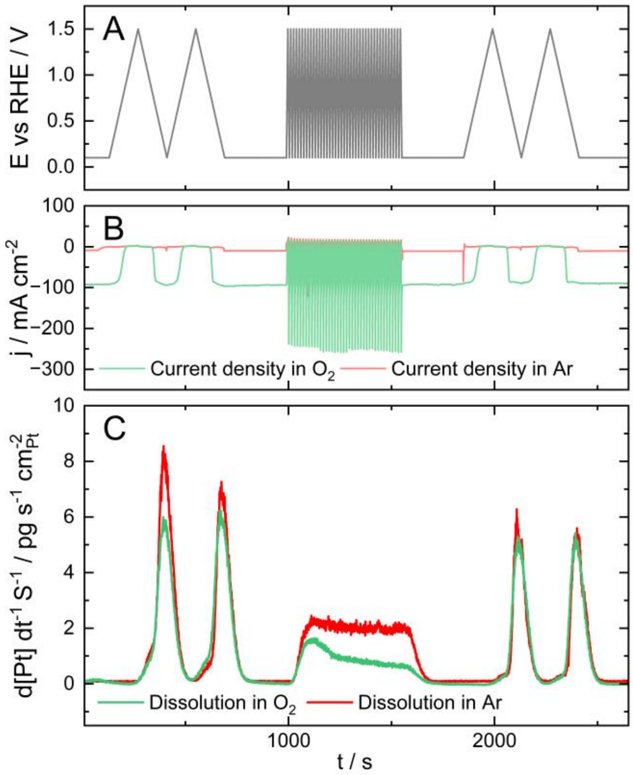

Figure S11: (A) The applied potential (B) the current density and (C) the dissolution profile as a function of time of dissolution study described in chapter 3.3 in the main text

9. Comparison of the Dissolved Pt Amount in the SFC, GDE-L and S-GDE

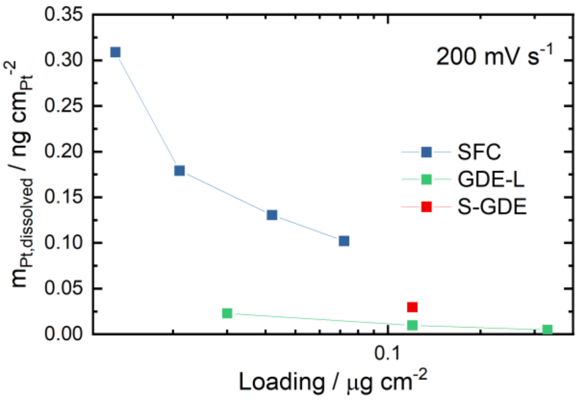

Figure S12: Comparing the dissolution amounts of the S-GDE to the GDL-L and the standard SFC during a CV with a scan rate of 200 mV s^{-1} as a function of loading. Data, besides S-GDE, from 1.

References

(1) Ehelebe, K.; Knöppel, J.; Bierling, M.; Mayerhöfer, B.; Böhm, T.; Kulyk, N.; Thiele, S.; Mayrhofer, K. J. J.; Cherevko, S. Platinum Dissolution in Realistic Fuel Cell Catalyst Layers. *Angewandte Chemie (International ed. in English)* **2021**, *60* (16), 8882–8888. DOI: 10.1002/anie.202014711.

(2) Ku, Y.-P.; Ehelebe, K.; Hutzler, A.; Bierling, M.; Böhm, T.; Zitolo, A.; Vorokhta, M.; Bibent, N.; Speck, F. D.; Seeberger, D.; et al. Oxygen Reduction Reaction in Alkaline Media Causes Iron Leaching from Fe-N-C Electrocatalysts. *Journal of the American Chemical Society* **2022**, *144* (22), 9753–9763. DOI: 10.1021/jacs.2c02088.
